# Casein Kinase 1 Delta (CK1δ) Regulates Period Length of the Mouse Suprachiasmatic Circadian Clock *In Vitro*

**DOI:** 10.1371/journal.pone.0010303

**Published:** 2010-04-22

**Authors:** Jean-Pierre Etchegaray, Elizabeth A. Yu, Premananda Indic, Robert Dallmann, David R. Weaver

**Affiliations:** 1 Department of Neurobiology, University of Massachusetts Medical School, Worcester, Massachusetts, United States of America; 2 Department of Neurology, University of Massachusetts Medical School, Worcester, Massachusetts, United States of America; Vanderbilt University, United States of America

## Abstract

**Background:**

Casein kinase 1 delta (CK1δ) plays a more prominent role in the regulation of circadian cycle length than its homologue casein kinase 1 epsilon (CK1ε) in peripheral tissues such as liver and embryonic fibroblasts. Mice lacking CK1δ die shortly after birth, so it has not been possible to assess the impact of loss of CK1δ on behavioral rhythms controlled by the master circadian oscillator in the suprachiasmatic nuclei (SCN).

**Methodology/Principal Findings:**

In the present study, mPER2::LUCIFERASE bioluminescence rhythms were monitored from SCN explants collected from neonatal mice. The data demonstrate that SCN explants from neonatal CK1δ-deficient mice oscillate, but with a longer circadian period than littermate controls. The cycle length of rhythms recorded from neonatal SCN explants of CK1ε-deficient mice did not differ from control explants.

**Conclusions/Significance:**

The results indicate that CK1δ plays a more prominent role than CK1ε in the maintenance of 24-hour rhythms in the master circadian oscillator.

## Introduction

In mammals, self-sustained circadian oscillators are present in most peripheral tissues and are coordinated by the master clock residing in the suprachiasmatic nuclei (SCN) of the anterior hypothalamic region [1]. At the molecular level, circadian oscillators are based on a molecular feedback loop that results in the cyclic expression of circadian genes and proteins over a period of approximately 24 hours. At the core of this 24-hour feedback cycle are the transcription activator complexes CLOCK:BMAL1 and NPAS2:BMAL1, which bind to E-box elements to activate transcription of their own negative regulators *Cryptochrome 1*, *Cryptochrome 2*, *Period 1* and *Period 2* (in mouse, abbreviated *mCry1*, *mCry2*, *mPer1*, and *mPer2*, respectively) [1], [2]. Upon translation, mCRY1-2 and mPER1-2 proteins accumulate in the cytoplasm and form mCRY:mPER repressor complexes that inhibit their own transcription by blocking the activity of nuclear CLOCK/NPAS2:BMAL1 complexes. As the concentration of mCRY1-2 and mPER1-2 proteins decreases, repression is relieved and a new cycle begins.

Phosphorylation of mPER proteins in the cytoplasm triggers their proteasome-dependent degradation [3]–[8]. The degradation of mPERs provides an important time-delay function, slowing the accumulation of mPER proteins and the formation of mCRY:mPER complexes. Several kinases including CK1 delta (CK1δ) and CK1 epsilon (CK1ε), casein kinase 2 (CK2), and glycogen synthase kinase 3-β (GSK3-β) can phosphorylate mPER proteins [3], [7], [9]–[12]. We recently demonstrated that mouse embryonic fibroblasts and liver tissues that are deficient in CK1δ, but not CK1ε, exhibit an approximately two-hour longer circadian period *in vitro* and concluded that CK1δ plays a more prominent role than CK1ε in maintaining the accuracy of the 24-hour circadian oscillator [9]. This conclusion is supported by the finding that a pharmacological inhibitor specific for CK1ε (PF-4800567) had a minimal effect on circadian period, while an inhibitor of both CK1δ and CK1ε (PF-670462) caused a large increase in period of oscillating fibroblasts [13]. Injections of PF-670462 induced large phase delays in the activity rhythms of rats and primates [14], [15]. Collectively, these results indicate that CK1δ plays a significant role in the regulation of circadian cycle length, while disrupting CK1ε has little or no effect on circadian period [9], [16]. However, this difference in the importance between the two kinases could be due to their relative expression levels, which might be tissue-specific. In this regard, the relative importance of CK1δ versus CK1ε in the SCN remains to be determined. The perinatal lethality of CK1δ deficiency precludes assessment of locomotor activity rhythms in this genotype. Therefore, to investigate the impact of CK1δ deficiency on SCN function, we isolated SCN explants from CK1δ-deficient neonatal mice and wild-type littermates, and monitored mPER2::LUC bioluminescence rhythms *ex vivo*. In parallel, studies were conducted on CK1ε-deficient mice. The results indicate differential roles of these two closely related kinases in the regulation of circadian period length in the SCN circadian clock.

## Materials and Methods

### Ethics Statement

All animal experiments were in accordance with NIH guidelines regarding the care and use of animals, and were reviewed and approved by the Institutional Animal Care and Use Committee of the University of Massachusetts Medical School (Protocols A-1315 and A-1572).

### Animals

Generation of mice with a targeted allele of casein kinase 1δ (*Csnk1d*), in which exon 2 is deleted (*CK1δ^Δ2/+^*), has been previously described [9]. Mice with this mutant allele are also referred to as B6.129S4-*Csnk1d^tm1.1Drw^*/J (Jackson Laboratories Stock# 010923). This allele is a functional null allele with respect to its ability to interact with, phosphorylate, or promote proteosomal degradation of PER proteins [9]. For bioluminescence experiments, neonatal mice bearing a single copy of the *Per2::luc* reporter gene were generated by overnight timed-breeding of mice heterozygous for the targeted allele of CK1δ, and with one of the mice having one or two alleles of *Per2::luc*. The *Per2::luc* reporter line is a “knock-in” line in which a portion of the *mPer2* locus has been replaced by the corresponding portion of *mPer2* in which the last coding exon is fused in-frame to firefly luciferase, and with an Simian virus 40 poly-adenylation signal to enhance expression [17]. Founder mice used to establish our colony of *Per2::luc* mice were generously supplied by Dr. Joseph S. Takahashi (University of Texas Southwestern Medical School, Dallas TX).

Pregnant mice were identified and singly housed before giving birth. Cages were checked for the presence of pups repeatedly on the day of expected birth, on gestational day (GD) 20 (where day 0.5 is the morning after separation from the male), and pups were generally collected within 2 hours of birth. In a few cases, pups were delivered by cesarean section on GD 20. Additionally, several experiments utilized mice (GD 20) that were homozygous for targeted disruption of CK1ε (*CK1ε^−/−^*) and bearing one copy of the *Per2::luc* reporter. Mice with this mutant allele, in which exons 2 and 3 have been deleted and the resulting transcript contains a stop codon in exon 4, are also referred to as B6.129S4-*Csnk1e^tm1.1Drw^*/J (Jackson Laboratories Stock# 010924). Genotyping of the mice from tail biopsies was performed using a PCR-based method, as previously described [9].

### SCN Explant Cultures

Neonatal mice were sacrificed by decapitation and brains were rapidly removed and placed in ice-cold Hank's balanced salt solution (HBSS). Brains were embedded in 2.5% low melting point agarose dissolved in HBSS and rapidly cooled on ice. Coronal brain sections of 400 µm were made using a vibratome and the section(s) containing the SCN were selected based on surrounding anatomical landmarks when viewed with a dissecting microscope. The SCN were then excised from the section using a scalpel blade, and were transferred to ice cold HBSS. Each SCN explant was placed on a sterile Millicell insert (Millipore, Billerica, MA, USA) and cultured according to the method described by Yamazaki and Takahashi [18]. Bioluminescence was monitored for 5 to 7 days on a Hamamatsu LM-2400 luminometer as described previously [9].

### Circadian Period Analysis

The mPER2::LUC bioluminescence rhythms from neonatal SCN explants were of low amplitude when compared to adult SCN tissues, damping over time and with a significant amount of noise. To extract the relatively weak circadian signal from the data, we employed wavelet transform. Wavelet transform is a suitable tool for analyzing non-stationary signals in the presence of noise. Furthermore, the wavelet method does not require any prior detrending or smoothing of the data. Our approach is similar to the method proposed by Price and colleagues [19]. We extracted the circadian period and amplitude by performing a time frequency decomposition of the experimental data using wavelet transform. The continuous wavelet transform of the recorded discrete data is obtained by the convolution of the data with a scaled and translated version of a mother wavelet [20]. We used complex-valued Morlet wavelet as the mother wavelet, as it is efficient in capturing the periodic waveforms from non-stationary data. At each instant of time, using a ridge extraction algorithm [21] we calculated the peak in the normalized scalogram. The period and the amplitude corresponding to this peak define the dominant component of the data.

It has to be noted that the exact characteristic of the noise in the bioluminescence profiles is not known. Furthermore, it has been reported that the correlated noise can produce quasi-periodic signals [22]. Hence to understand the effect of correlated noise, if any, on the data collected, we analyzed bioluminescence records from wells containing no tissue. Using our wavelet algorithm we explored the possibility of any quasi-periodicities in 20 “no-tissue control” profiles. We found the presence of low amplitude, quasi-periodic signals mostly outside the circadian range of 18–36 hours; these signals were most commonly in the range of 40–70 hours. However these signals were less robust in their rhythmicity and showed large variability in their period of oscillation. Since these quasi-periodic signals may appear as the dominant component when the circadian amplitude is lower than the quasi-periodic amplitude, we restricted our ridge extraction within the period range of 18–36 hours. The period and the amplitude corresponding to this peak are the circadian period and amplitude, respectively, for the mPER2::LUC bioluminescence rhythm.

To validate this method further, we analyzed mPER2::LUC bioluminescence rhythms from 15 adult tissue explants using both the modified wavelet method described above and a method we and others have used previously [9], [23]. Results from the two methods were highly correlated (r^2^ = 0.7789, P<0.0001).

**Figure 1:**
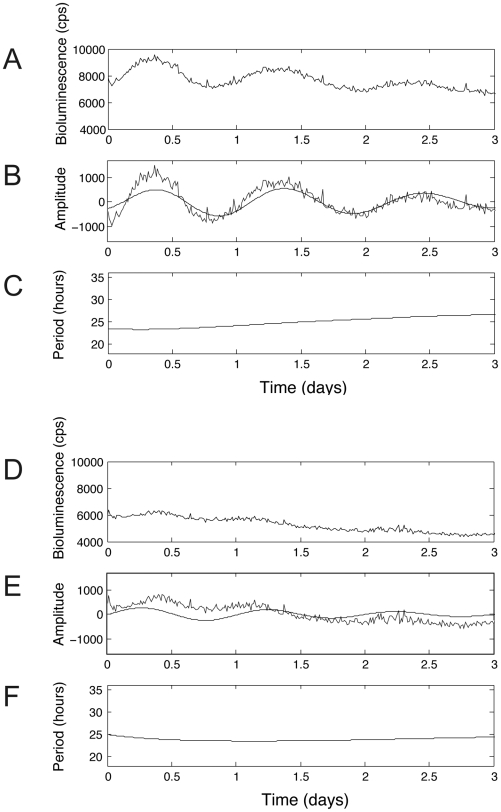
Representative mPER2::LUC bioluminescence profiles, illustrating methods for period estimation from neonatal SCN explants. (A) Representative example of a raw bioluminescence profile fulfilling the stringent criteria, which led to selection of 124 out of 235 mPER2::LUC bioluminescence profiles. (B) Extracted circadian amplitude (black sinusoidal line) along with the linear detrended representation (jagged black line) of the raw data shown in (A). (C) Period estimation from data shown in (A). (D) Representative example of a raw bioluminescence profile that was excluded using the stringent criteria. (E) Extracted circadian amplitude (black sinusoidal line) along with the linear detrended representation (jagged black line) of the raw data shown in (D). (F) Period estimation from data shown in (D). Amplitude and period were extracted from the data using wavelet and ridge extraction algorithms. The extracted amplitude for the profile in (A) shows robust oscillation (shown in (B)) with a sustained period value in the circadian range (shown in (C)). The extracted amplitude for the profile shown in (D) shows a weak oscillation, with the amplitude of the bioluminescence rhythm falling to less than 150 counts per second within the recording interval (shown in E). This record is one of 111 excluded by these criteria.

We analyzed 235 mPER2::LUC bioluminescence profiles of neonatal SCN explants derived from CK1δ-deficient, CK1ε-deficient and wild-type control mice. Many of the records from neonatal mice were not obviously rhythmic by eye; however, wavelet method was able to determine the periodicities from these data. To ensure a more stringent separation of signal from noise across the population of records, we instituted a set of criteria to select the profiles with robust oscillation for at least three days. The criteria were developed by having three observers, blind to genotype, determine whether a record of background-subtracted bioluminescence had sufficiently convincing rhythmicity for inclusion, and then determining the values that eliminated records that were consistently scored as “not rhythmic” and which eliminated those records lacking tissue. The instantaneous variation of period and the amplitude of rhythmicity in the circadian range were examined in developing the criteria, and then the criteria were applied to all records. Only those profiles that (1) exhibited consistent period in the circadian range (18–36 hours) for three days and (2) had amplitude of oscillation whose magnitude was greater than 150 bioluminescence units (counts per second [cps]) for three days were included. Using these criteria, 124 out of the 235 mPER2::LUC bioluminescence profiles were selected (Figure 1). Analysis of these 124 selected profiles led to the same conclusions as were found with the whole set of 235 profiles (see below).

### Statistical Analyses

Period values derived from bioluminescence recordings of neonatal SCN explants were compared between genotypes using parametric statistical methods (GraphPad Prism Software, La Jolla, CA, USA). The data were assessed for equality of variance; in one case where the variance was unequal between the groups, the subsequent t-test was conducted using Welsh's correction. More specifically, period values from SCN explants derived from *CK1δ^Δ2/Δ2^*, *CK1δ^Δ2/+^* and wild-type control mice were analyzed by one-way ANOVA and Tukey's multiple comparison test. Period values of bioluminescence rhythms from neonatal SCN explants derived from CK1ε-deficient and wild-type control mice were analyzed by unpaired t-test with Welch's correction. Statistical significance was defined as *P*<0.05. Data are reported as mean ± SEM.

## Results

**Figure 2:**
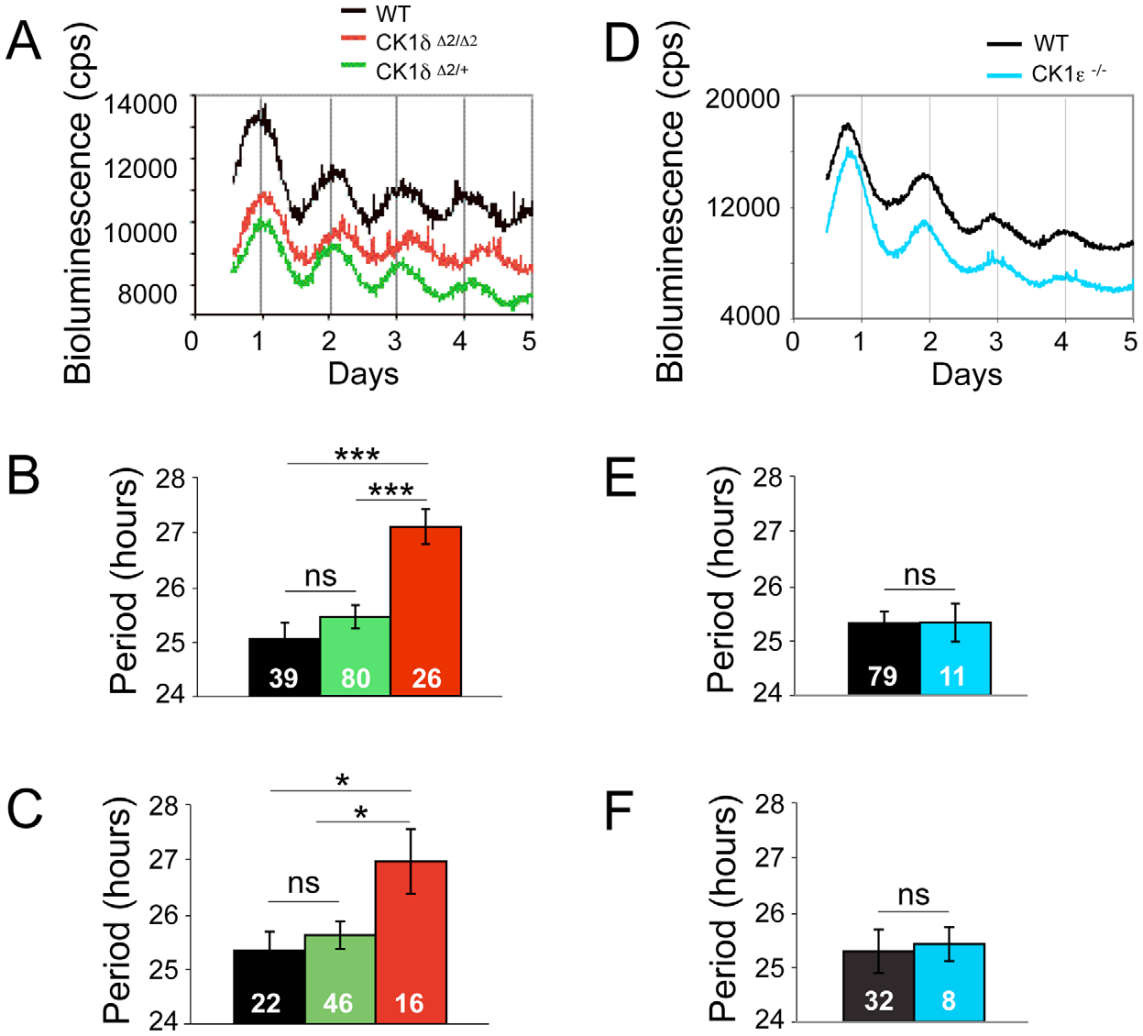
Neonatal SCN explants deficient in CK1δ, but not CK1ε, exhibit longer circadian period of mPER2::LUC bioluminescence. (A) Representative bioluminescence profiles of neonatal SCN explants derived from *CK1δ^Δ2/Δ2^*, *CK1δ^Δ2/+^* and wild-type mice. (B) Period analysis of bioluminescence profiles from all *CK1δ^Δ2/Δ2^*, *CK1δ^Δ2/+^* and wild-type SCN explants studied. (C) Period analysis of bioluminescence profiles from those *CK1δ^Δ2/Δ2^*, *CK1δ^Δ2/^* and wild-type SCN explants that fulfilled the stringent criteria for inclusion. (D) Representative bioluminescence profiles of neonatal SCN explants derived from *CK1ε^−/−^* and wild-type mice. (E) Period analysis of bioluminescence profiles from all *CK1ε^−/−^* and wild-type SCN explants studied. (F) Period analysis of bioluminescence profiles from those *CK1ε^−/−^* and wild-type SCN explants meeting the stringent criteria for inclusion. In Panels B, C, E and F, values represent the mean ± SEM and sample sizes are indicated within each bar. Asterisks indicate significant differences (* indicates *P*<0.03; *** indicates *P*<0.0001), and ‘ns’ indicates no significant difference (*P*>0.05).

Bioluminescence rhythms from SCN explants lacking functional CK1δ (*CK1δ^Δ2/Δ2^*) had a longer circadian period (27.09±0.32 {Mean ± SEM} hours) than SCN explants derived from wild-type (*CK1δ^+/+^*; 25.04±0.29 hours) or heterozygous (*CK1δ^Δ2/+^*; 25.44±0.21 hours) neonatal mice (*P*<0.0001; Figure 2A, 2B). In contrast, there was no significant difference (*P*>0.9) in the period length of mPER2::LUC bioluminescence rhythms between neonatal SCN explants derived from *CK1ε^−/−^* (25.31±0.34 hours) and wild-type controls (25.30±0.22 hours) (Figure 2D, 2E).

The amplitude of mPER2::LUC bioluminescence rhythms from neonatal SCN explants was low, making period estimation more difficult than in adult tissues. Therefore, in addition to analyzing the entire dataset, we used a set of more stringent criteria to select the bioluminescence profiles that exhibited more stable and robust circadian oscillations. The analysis of profiles selected with these criteria gave the same pattern of results as with the unselected profiles. More specifically, there was a significant lengthening of circadian periodicity (*P*<0.03) in SCN explants derived from *CK1δ^Δ2/Δ2^* neonates when compared to heterozygous *CK1δ^Δ2/+^* or wild-type littermates (Figure 2C). Furthermore, no significant difference was found in period length between *CK1ε^−/−^* neonatal SCN explants and explants from wild-type controls (Figure 2F).

## Discussion

The present results demonstrate that CK1δ plays a more prominent role than CK1ε in maintaining the period length of circadian rhythmicity in the SCN. This same difference in relative importance of the two closely related kinases has been reported for peripheral tissues (liver and embryonic fibroblasts; [9]). Furthermore, the period length of SCN-generated behavioral rhythms is unaffected in *CK1ε^−/−^* mice [9], [16]. These findings seem consistent with two interpretations. First, CK1ε may be irrelevant to period regulation in the circadian oscillator. Alternatively, both CK1δ and CK1ε may play an important role in the circadian clock, with the role of CK1δ being more prominent in loss-of-function studies, perhaps because of higher levels of expression of CK1δ (as recently reported for fibroblasts; [24]).

Recent work by others strongly supports the latter interpretation, invoking partial functional redundancy between the kinases. Using a pharmacological approach, Walton *et al.*, [13] showed that an inhibitor of both CK1δ and CK1ε (PF-670462) lengthened the period of mPER2::LUC bioluminescence rhythms of rat fibroblasts in a dose-dependent manner, reaching periods up to 35 hours. In contrast, an inhibitor with relative selectivity for CK1ε (PF-4800567) did not affect circadian period, except at high concentrations that could also affect CK1δ. The simplest explanation, i.e. the effect of PF-670462 on circadian period is due to inhibition of CK1δ alone, is not consistent with our results from a genetic model, as we see only a 2-hour increase in period in CK1δ-deficient liver, fibroblasts, and SCN ([9]; present results) rather than a 12-hour increase. An alternative explanation is that inhibition of both kinases is needed to see large effects on period. We have been unable to test this idea due to limitations in our ability to generate animals or tissues with simultaneous disruption of both kinases. This idea is supported, however, by recent studies examining the effects of over-expression of a dominant-negative version of CK1ε [24]. Over-expression of this dominant-negative construct reduced the activity of both CK1δ and CK1ε, and caused a modest increase in period in wild-type fibroblasts. Remarkably, however, this construct completely disrupted circadian rhythms of mPER2::LUC bioluminescence in CK1δ-deficient fibroblasts [24], further suggesting redundant roles of CK1δ and CK1ε in maintaining 24-hour oscillations. Furthermore, in a recent small-molecule screen, Isojima and colleagues found that a common feature of compounds causing an increase in circadian period was inhibition of CK1δ/CK1ε activity, and RNA interference targeting either kinase lengthened period in human U2 osteosarcoma cells, although reducing expression of CK1δ by RNA interference had a larger effect than knocking down CK1ε [25].

These results suggest that CK1δ-deficient tissues remain rhythmic, albeit with a longer period, due to the activity of CK1ε. To confirm this apparent functional redundancy between these two kinases in the circadian system, it would be ideal to use a genetic approach to simultaneously disrupt the expression of both CK1δ and CK1ε. Complementary experiments combining genetic and pharmacological approaches to investigate the relative role of the two kinases are also needed.
